# Vascular Disruptive Hydrogel Platform for Enhanced Chemotherapy and Anti-Angiogenesis through Alleviation of Immune Surveillance

**DOI:** 10.3390/pharmaceutics14091809

**Published:** 2022-08-28

**Authors:** Fasheng Li, Xinmei Shao, Dehui Liu, Xiaogang Jiao, Xinqi Yang, Wencai Yang, Xiaoyan Liu

**Affiliations:** 1 Department of Imaging, The Fifth Affiliated Hospital of Jinan University, Jinan University, Heyuan 517000, China; 2 Department of Neurology, The Fifth Affiliated Hospital of Jinan University, Jinan University, Heyuan 517000, China; 3 Department of Interventional, The Fifth Affiliated Hospital of Jinan University, Jinan University, Heyuan 517000, China

**Keywords:** anti-angiogenesis therapy, vascular disruption, immunogenic cell death, immune surveillance

## Abstract

Patients undergoing immunotherapy always exhibit a low-response rate due to tumor heterogeneity and immune surveillance in the tumor. Angiogenesis plays an important role in affecting the status of tumor-infiltrated lymphocytes by inducing hypoxia and acidosis microenvironment, suggesting its synergistic potential in immunotherapy. However, the antitumor efficacy of singular anti-angiogenesis therapy often suffers from failure in the clinic due to the compensatory pro-angiogenesis signaling pathway. In this work, classic injectable thermosensitive PLGA-PEG-PLGA copolymer was used to construct a platform to co-deliver CA4P (vascular disruptive agent) and EPI for inducing immunogenic cell death of cancer cells by targeting the tumor immune microenvironment. Investigation of 4T1 tumor-bearing mouse models suggests that local administration of injectable V+E@Gel could significantly inhibit the proliferation of cancer cells and prolong the survival rate of 4T1 tumor-bearing mouse models. Histological analysis further indicates that V+E@Gel could effectively inhibit tumor angiogenesis and metastasis by down-regulating the expression of CD34, CD31, MTA1 and TGF-β. Moreover, due to the sustained release kinetics of V+E@Gel, its local administration relieves the immune surveillance in tumor tissues and thus induces a robust and long-lasting specific antitumor immune response. Overall, this work provides a new treatment strategy through the mediation of the tumor immune microenvironment by vascular disruption to fulfill enhanced chemotherapy and immunotherapy.

## 1. Introduction

Compared to conventional chemotherapy and surgery, immunotherapy is emerging as an alternative strategy for the treatment of various types of cancer [1,2,3,4]. Current immunotherapies comprise checkpoint blockade therapy, cancer vaccines and adoptive T-cell transfer therapy. Thus far, patients treated with immunotherapy generally exhibit a rather low response rate, ranging from 5 to 30% [5,6,7,8]. The main reasons for the low therapeutic efficacy include the tumor heterogeneity among different patients, and the immune surveillance environment in human bodies, containing immunosuppressive cells, including myeloid-derived suppressor cells (MDSCs), tumor-associated macrophages (TAMs) and regulatory T cells (Tregs) [9,10,11,12]. Therefore, it would be a good strategy to improve immunotherapy efficacy by relieving immune surveillance.

Anti-angiogenesis therapy has become a burgeoning strategy in cancer treatment because of its high efficacy in ablating solid tumors [13,14]. Angiogenesis takes a critical role in various physiological processes, such as wound healing and organ or embryonic development [15]. Moreover, angiogenesis participates in the growth and metastasis of tumors by providing abundant oxygen and nutrients [16,17]. It is known that hypoxia and acidosis in the tumor core are attributed to the disproportional blood supply, which always leads to the imbalance of local pro-angiogenic factors and anti-angiogenic factors such as vascular endothelial growth factor (VEGF), etc. [18,19,20]. Importantly, anti-angiogenesis therapy is designed to cut off the blood nutrient supply in a tumor and thus efficiently enhance its sensitivity to other clinical therapies [21]. Notably, angiogenesis often affects the status of tumor-infiltrating lymphocytes [22]. To be more specific, abnormal angiogenesis would cause high interstitial fluid pressure and deficiency of adhesive molecules such as vasculature cell adhesion molecule-1 (VCAM-1), which further decreases the number of tumor-infiltrated lymphocytes (TIL) [23,24]. Furthermore, the hypoxia microenvironment in the tumor core could upregulate the expression of chemokine (C-C motif) ligand-22 and the polarization of tumor-associated macrophage (TAM) to M2-like phenotype, which consequently undermines the functions of TIL [25,26,27,28]. Therefore, anti-angiogenesis therapy holds the synergistic potential to promote the therapeutic efficacy of immunotherapy. However, previous studies showed that singular anti-angiogenesis therapy generally shows limited efficacy in patients bearing cancers [25,29,30,31]. As combination therapy, which simultaneously delivers different drugs with independent therapeutic mechanisms, would greatly boost treatment outcomes with lowered therapeutic threshold dosage and negligible drug resistance, the co-delivery of vascular disruption agents and chemotherapeutic drugs to fulfill enhanced chemotherapy and immunotherapy holds great potential for efficient cancer treatment. 

Epirubicin (EPI), with a similar structure to doxorubicin, is a classic chemotherapy drug that is widely used in breast cancer treatment [32,33]. Its antitumor mechanism is literally realized through its intercalation in DNA structure and its inhibitive effect on topoisomerase II with consequent interference with DNA or RNA synthesis. EPI was also proven to be able to inhibit the Forkhead box protein p3 (Foxp3), the representative indicator of Treg cells. Combretastatins are stilbene derivatives that are widely used as vascular disruptive agents (VDA) in cancer therapy. Their specific bind with tubulin in endothelial cells could effectively inhibit the polymerization of tubulin, thus destroying endothelial cells, leading to the leakage of blood vessels, and consequently inducing tumor necrosis [34]. Moreover, clinical trials demonstrated that their combination with chemotherapeutics drugs such as cisplatin and paclitaxel could reduce the dosage of chemotherapeutics drugs and effectively alleviate the side effects or toxicity [35]. Therefore, we proposed that co-delivery of EPI and vascular disruption agents to tumor tissues may effectively relieve the immune surveillance and induce a robust, specific cytotoxic immune response.

**Scheme 1:**
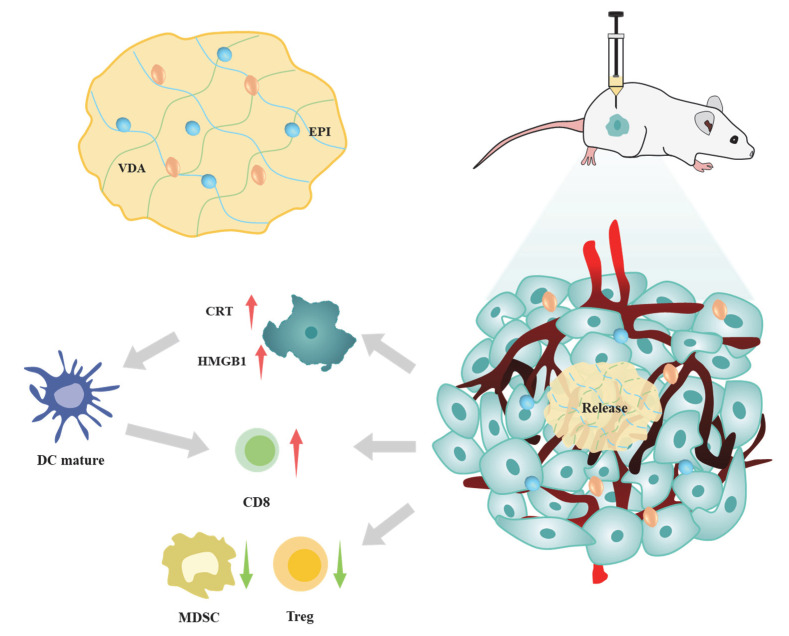
Schematic illustration of V+E@Gel on promoting chemotherapy and anti-angiogenesis therapy through relieving immune surveillance. Local administration of V+E@Gel could promote the infiltration of CD8+ lymphocytes and decrease the number of MDSCs and Tregs, relieve the immune surveillance in tumor tissues and thus induce robust and long-lasting immunogenic cell death.

Local delivery of chemotherapeutic drugs based on the injectable hydrogel has become a promising drug-delivery platform applied for various cancer treatments [36,37,38,39,40]. Due to its great histocompatibility, biodegradability, and tunable mechanical or viscoelastic properties, injectable hydrogel formulations were approved in clinical trials. Through intramolecular covalent or non-covalent interaction between polymers, hydrogel could control the release of laden drugs better in a more sustainable manner and prevent the overactivation of the immune system, which would efficiently alleviate the side effects or toxicity due to high dosage, make immunotherapies more tolerable and thus improve its therapeutic efficacy [41,42,43]. Additionally, its favorable mechanical and viscoelastic property also allows the hydrogel to be a tissue scaffold, which could cultivate an immunogenic niche locally through recruiting host endogenous immune cells for reshaping the tumor immune microenvironment [44]. In this work, we started from the design and synthesis of the hydrogel delivery system, which is composed of classic injectable thermosensitive PLGA-PEG-PLGA copolymer co-loaded with CA4P (vascular disruptive agent) and EPI for inducing immunogenic cell death of cancer cells (Scheme 1). Furthermore, we investigated the antitumor efficacy of V+E@Gel on 4T1 tumor-bearing mouse models. In addition, its effect on inducing immunogenic cell death (ICD) and relieving the immune surveillance in tumor tissues was investigated by a flow cytometer either. We believe this work may provide a new treatment strategy through the mediation of the tumor immune microenvironment by vascular disruption to fulfill enhanced anti-angiogenesis and chemotherapy. 

## 2. Materials and Methods

### 2.1. Materials

Lactic acid (LA), glycolic acid (GA), stannous octoate, hydroxyl-terminated PEG, combretastatin A4 disodium phosphate (CA4P) and epirubicin (EPI) were purchased from Sigma–Aldrich (USA). Primary antibodies, including anti-PCNA, anti-CD34, anti-CD31, anti-MTA1, anti-TGF-β, anti-CRT and anti-HMGB1, were obtained from Abcam (Cambridge, UK). Antibodies for flow cytometric analysis, including anti-CD45, anti-CD11c, anti-CD86, anti-CD11b, anti-Gr-1, anti-CD3, anti-CD4, anti-CD8 and anti-FOXP3 were purchased from Biolegend. The 4′,6-diamidino-2-phenylindole (DAPI) and TUNEL assay kit were purchased from Thermo Fisher Scientific, Waltham, MA, USA.

### 2.2. Synthesis of PLGA-PEG-PLGA

Thermosensitive triblock copolymer, PLGA-PEG-PLGA, was synthesized through ring-opening polymerization, as previously described [45]. Briefly, hydroxyl-terminated PEG was firstly stirred for 4 h at 150 °C under vacuum. Then, LA and GA were added into the above solution at a molar ratio of 8:1, followed by dehydration at 80 °C under argon protection for 3 h. After all the monomers melted, 0.2 wt % stannous octoate was added to initiate the polymerization, and the reaction was stirred at 150 °C for 12 h. The unreacted monomers and toluene were removed under vacuum at 120 °C. The final product was washed with 80 °C water three times and lyophilized for further use.

### 2.3. Preparation and Characterization of V+E@Gel

For the preparation of blank hydrogel, VDA@Gel, EPI@Gel, or V+E@Gel, 20% wt PLGA-PEG-PLGA copolymer solution (PBS buffer) was first prepared and kept under 4 °C. VDA (2 mg/mL) or EPI (1.6 mg/mL) were then dispersed in the stock solution and kept in the fridge for further use. Hydrogel formulations were then prepared in an oven at 37 °C to allow sol–gel transition of PLGA-PEG-PLGA. The as-prepared blank hydrogel was characterized by cryogenic scanning electron microscopy (cyro-SEM) (HITACHI, SU 8020, Hitachi, Japan), and lower critical solution temperature (LCST) of blank hydrogel and V+E@Gel was detected by MARS60 (HAAKE, Karlsruhe, Germany).

### 2.4. In Vitro Release of V+E@Gel

The release behavior of VDA and EPI in the hydrogel was investigated by High-Performance Liquid Chromatography (HPLC) and UV-Vis spectrometer. An amount of 1 mL V+E@Gel was immersed in PBS buffer (pH 7.4) at 37 °C, during which 1 mL sample was collected at specific time intervals. The concentration of VDA in the collected samples was determined by HPLC at 305 nm using Agilent 1260 Infinity II liquid chromatography system with an Agilent Eclipse Plus C18 column (250 mm × 4.6 mm, inside diameter (i.d.), particle size 5 μm) (Agilent Technologies, Santa Clara, CA, USA). The mobile phase was set as 50% A: 0.05mol/L potassium dihydrogen phosphate; 50% B: methanol + acetonitrile (*v*/*v*: 80:20) at a flow rate of 1 mL/min. The concentration of EPI in the collected samples was determined by UV-Vis spectrometer at 480 nm. 

### 2.5. In Vivo Antitumor Efficacy of V+E@Gel

The antitumor efficacy of V+E@Gel was evaluated on 4T1 tumor-bearing mouse models. The 4T1 tumor-bearing mouse models were established by subcutaneous injection of 2 × 10^6^ 4T1 cells in BALB/c mice. Mice with tumor volume that reached 100 mm^3^ were chosen for the following experiments. The 4T1 tumor-bearing mouse models were then randomly divided into 5 groups: (1) Control group: intratumoral injection (i.t.) of 100 µL saline solution; (2) Blank hydrogel group: i.t. injection of blank hydrogel; (3) VDA@Gel group: *i.t.* injection of VDA@Gel (100 µL, 200 µg VDA); (4) EPI@Gel group: i.t. injection of EPI@Gel (100 µL, 160 µg EPI); (5) V+E@Gel group: i.t. injection of V+E@Gel (100 µL, equivalence of 200 µg VDA and 160 µg EPI). The whole evaluation lasted for 15 days, during which tumor volume, body weight, and body temperature of mice from each group were monitored every 3 days. Then, all mice were euthanized, and tumors from each group were collected for H&E staining, TUNEL assay, immunofluorescence analysis (CD31 and TGF-β), immunohistochemical analysis (PCNA, CD34, MTA1) and flow cytometric analysis.

Another bunch of mice performed as the above description and were monitored for 60 days to evaluate the potential of V+E@Gel to prolong the survival rate of 4T1 tumor-bearing mouse models.

### 2.6. Histological Analysis of Tumor Slices

For immunofluorescence analysis, tumor slices were dewaxed and rehydrated in PBST. Antigens were then retrieved through 45 s heating in a microwave oven. After cooling down, tumor slices were mounted with 3% hydrogen peroxide for 5 min to inactivate endogenous peroxidase, followed by a rinse of PBST. Tumor slices were then blocked with 10% goat serum for 40 min and then incubated with corresponding primary antibodies (anti-CD31, anti-TGF-β, anti-CRT and anti-HMGB1) antibodies at 4 °C overnight. Tumor slices were finally rinsed with PBST and mounted for further detection under confocal microscopy (Leica SP8). The semi-quantitative analysis was performed on Image-Pro Plus 6.0.

For immunohistochemical analysis, tumor slices first proceeded in a similar manner to the immunofluorescence analysis. After blocking with 10% goat serum for 40 min, tumor slices were mounted with corresponding primary anti-PCNA, anti-CD34 and anti-MTA1 at 4 °C overnight. After rinsing with PBST three times, the heart slices were covered with secondary antibody and incubated at room temperature for 60 min. Tumor slices were rinsed, stained with DAB substrate and hematoxylin, and finally mounted for further detection via Vectra Automated Quantitative Pathology Imaging System (PerkinElmer, Waltham, MA, USA). Semi-quantitative analysis was performed on ImageJ software.

### 2.7. Toxicity of V+E@Gel

The toxicity of V+E@Gel was investigated on BALB/c mice for a month. Briefly, BALB/C mice were s.c. injected with 100 µL V+E@Gel and then fed for 30 days, during which mice were euthanized, and blood samples and major organs were collected on days 5, 15 and 30. Organ slices, including lung, liver, spleen, kidney and heart, were stained with hematoxylin and eosin according to the instruction of the manufacturer. Biochemical indices in blood samples, including alanine aminotransferase (ALT), creatinine (CREA), uric acid (UA), total protein (TP), blood urea nitrogen (BUN) and aspartate transaminase (AST), were determined by an automatic biochemical analyzer.

### 2.8. Statistical Analysis

All values are expressed as mean ± standard deviation (SD). Comparisons among all groups were evaluated using one-way ANOVA or Student’s *t*-test by GraphPad Prism version 8.0 for Windows (GraphPad Software, San Diego, CA, USA), and data with *p* < 0.05, *p* < 0.01, or *p* < 0.001 were considered statistically significant.

## 3. Results

### 3.1. Preparation and Characterization of V+E@Gel

**Figure 1:**
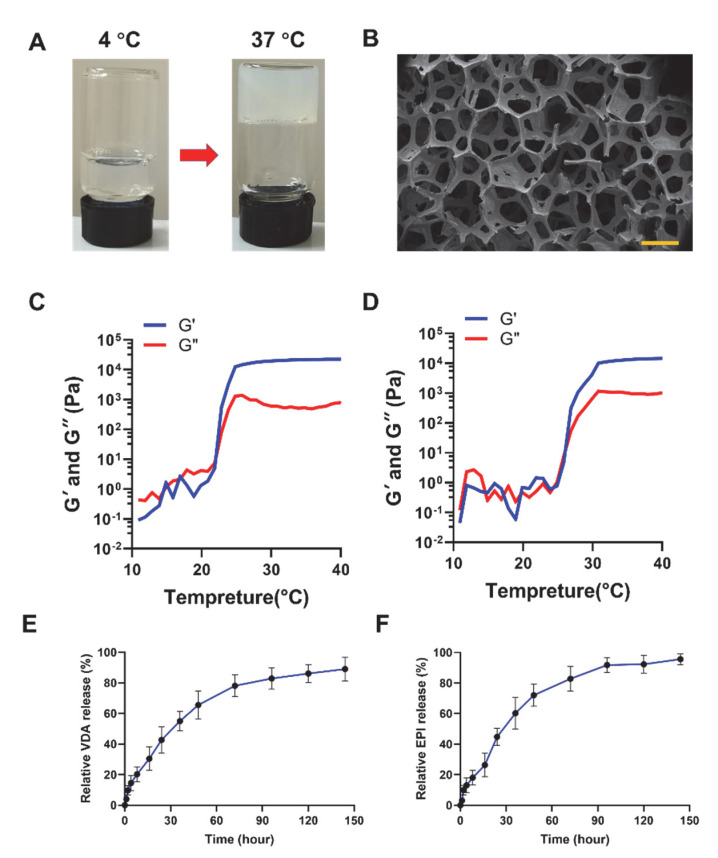
Preparation and characterization of V+E@Gel. (**A**) Representative photos of sol–gel transition of PLGA-PEG-PLGA hydrogel; (**B**) Morphological study of V+E@Gel by cryo-SEM, scale bar = 500 µm; Dynamic mechanical analysis of (**C**) blank hydrogel and (**D**) V+E@Gel; In vitro release kinetics of (**E**) VDA and (**F**) EPI in V+E@Gel. All data are shown as mean ± S.D. (*n* = 3).

PLGA, as a type of synthetic polyester, has been widely applied in various kinds of drug delivery in different disease treatments due to its high biocompatibility and biodegradability. Through conjugation with a hydrophilic segment of PEG, PLGA exhibited thermosensitive properties at physiological temperatures. Compared with the other stimuli-responsive hydrogel, the thermosensitive property of PLGA-PEG-PLGA is tunable in a wide temperature range based on the optimization of segments. Additionally, as exogenous stimuli, the temperature is more facile, accessible and tunable than the other stimuli, such as pH or GSH, which thus make it an ideal platform for drug delivery of biomacromolecules such as lysozyme, porcine growth hormone (pGH), granulocyte colony-stimulating factor, insulin and recombinant hepatitis B surface antigen [46,47]. Therefore, based on the ring-open polymerization, a thermosensitive PLGA-PEG-PLGA triblock copolymer was synthesized according to the previous protocol. The sol–gel transition of PLGA-PEG-PLGA was firstly investigated under 37 °C, as shown in Figure 1A. CA4P and EPI co-loaded hydrogel (V+E@Gel) was then prepared based on the self-assembly of PLGA-PEG-PLGA under physiological relevant temperature. The results of cryo-SEM (Figure 1B) demonstrated that the as-prepared V+E@Gel was exhibited as a 3D porous structure with a diameter of ~500 µm, which enabled the high loading of CA4P and EPI. LCST, as the critical index of the thermosensitive copolymer, was also analyzed by rheometer. As shown in Figure 1C,D, the LCST of PLGA-PEG-PLGA was around 22 °C while the LCST of V+E@Gel slightly increased to −26 °C, suggesting the addition of CA4P and EPI did not influence the thermosensitive property of PLGA-PEG-PLGA.

Before applying in the animal experiments, the release kinetics of V+E@Gel was firstly investigated in vitro based on the analysis of HPLC and UV-Vis spectrometer. As detected in Figure 1E,F, the burst release of VDA and EPI in the first 24 h could be maintained under 20%, which was significantly contained in comparison with the other hydrogel formulations based on PF127, hyaluronic acid, or methoxy poly (ethylene glycol)-poly (γ-ethyl-L glutamate) (mPEG-b-PELG) [48,49]. This sustained-release kinetics may effectively avoid the acute side effect or toxicity induced by high dosages of these chemotherapeutics. Moreover, the release phase of both drugs could be prolonged to 150 h, holding the potential to continuously trigger the immune reaction in tumor tissues. Overall, this injectable thermosensitive hydrogel formulation with sustained release kinetics may be applicable to cancer treatment. This section is divided into subheadings. It should provide a concise and precise description of the experimental results, their interpretation and the experimental conclusions that can be drawn.

### 3.2. In Vivo Antitumor Efficacy of V+E@Gel

**Figure 2:**
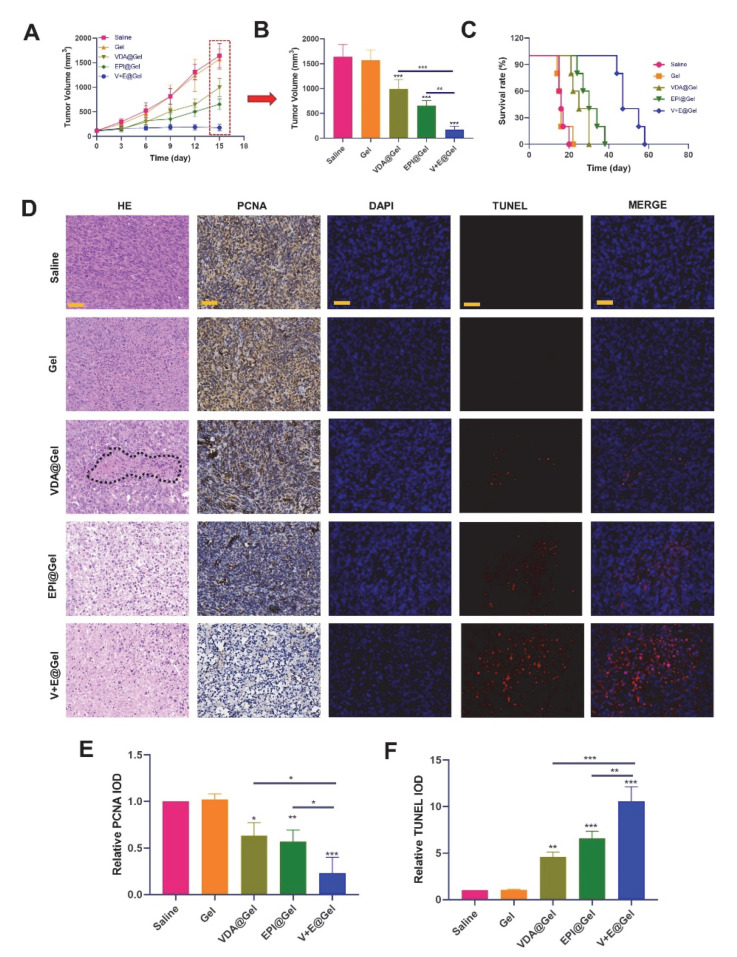
In vivo antitumor efficacy of V+E@Gel. (**A**) Change in tumor volume from each treatment group during 15 days; (**B**) Relative tumor volume from each treatment group on day 15; (**C**) Survival rate of mice from each treatment group during 60 days evaluation; (**D**) Histological analysis of tumor slices from each treatment group by H&E staining, immunohistochemical analysis and TUNEL assay, scale bar = 50 µm; Semi-quantitative analysis of (**E**) PCNA expression and (**F**) TUNEL expression in tumor tissues. All data are shown as mean ± S.D. (*n* = 5), * *p* < 0.05, ** *p* < 0.01, and *** *p* < 0.001 vs. Saline.

Antitumor efficacy of V+E@Gel was thus investigated on 4T1 tumor-bearing mouse models. It could be seen in Figure 2A,B and Figure S1 that after being treated with VDA@Gel, EPI@Gel or V+E@Gel for 15 days, mice tumor volume was reduced by ~40%, 60% and 92%, respectively, compared with the saline group. While in the gel group, the tumor volume was comparable to the saline group, indicating the antitumor efficacy of V+E@Gel was mostly derived from the laden drugs. The survival rate from each treatment group (Figure 2C) further demonstrated the great antitumor efficacy of V+E@Gel as the mice survival rate from the V+E@Gel group could be maintained at 100% for more than 40 days, while in the other groups, the survival rate dropped down to 0% on day 40.

Histological analysis, including H&E staining, immunohistochemical analysis and TUNEL assay, was also performed to evaluate the apoptosis in tumor tissues. As observed in H&E staining (Figure 2D), necrosis (circled by a black dotted line) could be observed in tumor tissues of the VDA@Gel group, indicating the decrease in tumor volume in the VDA@Gel group was derived from the vascular disruptive effect of combretastatin (CA4P). In contrast, in both the saline and gel groups, necrosis could hardly be observed. Moreover, in contrast with the saline group, purple colors indicating the proliferation of cancer cells was significantly decreased in the V+E@Gel group, preliminarily proving the inhibitive effect of V+E@Gel on the proliferation of cancer cells. The proliferation status of cancer cells was further confirmed by the detection of proliferating cell nuclear antigen (PCNA) through immunohistochemical analysis. As shown in Figure 2D,E, in contrast with the saline group, the IOD value of PCNA from VDA@Gel, EPI@Gel and V+E@Gel groups was, respectively, down-regulated by 40%, 50%, and 80%. Correspondingly, the apoptosis in tumor tissues was significantly enhanced, as the IOD value of TUNEL from VDA@Gel, EPI@Gel and V+E@Gel groups was significantly increased by 3-, 5-, and 9-fold, respectively. In addition, mice body weight and temperature from VDA@Gel, EPI@Gel and V+E@Gel groups were all maintained steady (Figures S2 and S3), preliminarily demonstrating the biocompatibility of PLGA-PEG-PLGA. Therefore, it could be concluded that local administration of injectable V+E@Gel may effectively inhibit the proliferation of cancer cells and prolong the survival rate of 4T1 tumor-bearing mouse models.

**Figure 3:**
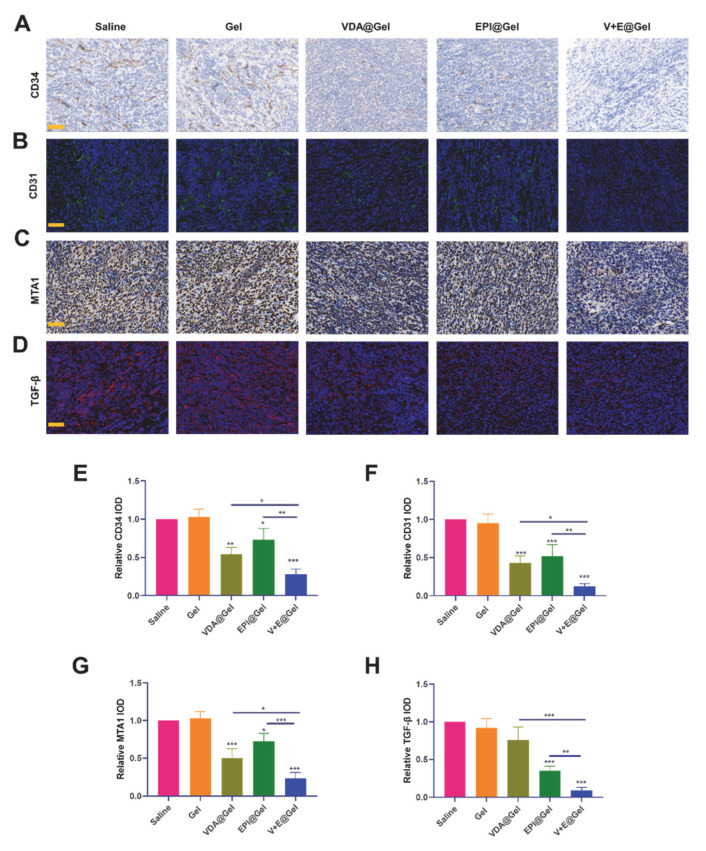
Anti-metastasis efficacy of V+E@Gel. Expression of (**A**) CD34, (**B**) CD31, (**C**) MTA1 and (**D**) TGF-β in tumor slices from each treatment group, scale bar = 50 µm; Semi-quantitative analysis of (**E**) CD34, (**F**) CD31, (**G**) MTA1 and (**H**) TGF-β expression in tumor slices from each treatment group. All data are shown as mean ± S.D. (*n* = 3), * *p* < 0.05, ** *p* < 0.01, and *** *p* < 0.001 vs. Saline.

Tumor metastasis is a leading reason for the high mortality of various types of cancer. The anti-metastatic potential of V+E@Gel was thus investigated based on histological analysis. As observed in Figure 3A,B,E,F, the expression of CD34 and CD31, an indicator of angiogenesis, were both significantly decreased in the V+E@Gel group. It is noteworthy that in the EPI@Gel group, the expression of CD34 and CD31 was a little higher than that in the VDA@Gel group. This may be due to the target of EPI being mainly focused on topoisomerase instead of vascular epithelial cells. Metastasis-associated protein1 (MTA1) is an indispensable factor that participates in tumor metastasis. Therefore, MTA1 expression and TGF-β level were both detected. As observed in Figure 3C,G, MTA1 expression in tumor tissues from the V+E@Gel group was significantly reduced by 80% in comparison with the saline group. Additionally, MTA1 expression in tumor tissues from the VDA@Gel group was also reduced by 50% in comparison with the saline group, demonstrating the inhibition of tumor metastasis and vascular disruptive effect of CA4P. In addition, in contrast with the saline group, the TGF-β level (Figure 3D,H), which represents tumor metastasis, was also, respectively, reduced by 30%, 60% and 90% in VDA@Gel, EPI@Gel and V+E@Gel group. Overall, intratumoral injection of V+E@Gel not only inhibits tumor growth but also effectively inhibits tumor angiogenesis and metastasis, which may be applicable in the clinical treatment of breast cancer.

### 3.3. Immunogenic Cell Death Induced by V+E@Gel

**Figure 4:**
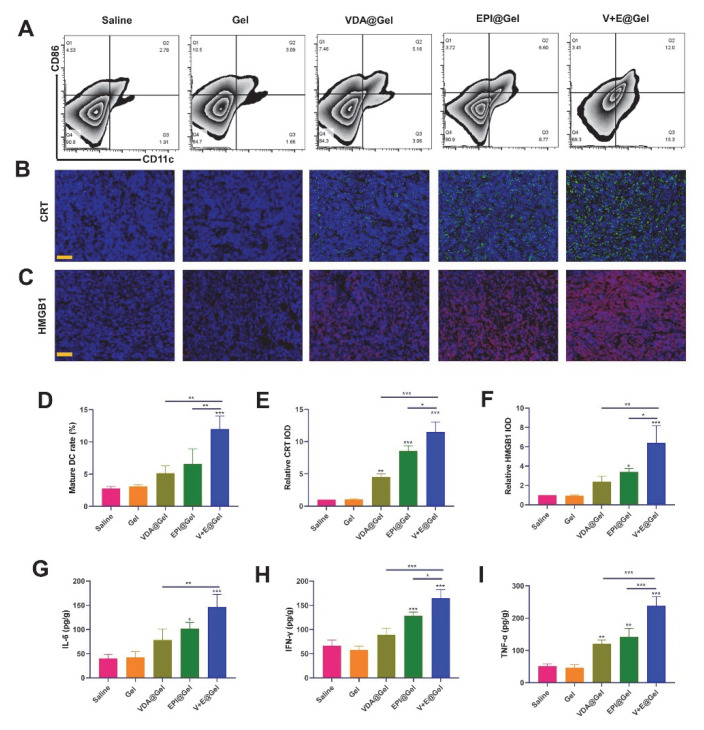
V+E@Gel activated the immunogenic cell death. (**A**) Flow cytometric analysis of infiltrated lymphocytes in tumor-draining lymph nodes; Immunofluorescence analysis of (**B**) CRT and (**C**) HMGB1 expression in tumor tissues from each treatment group, scale bar = 50 µm. (**D**) Quantitative analysis of mature DC cells in tumor tissues from each treatment group. Semi-quantitative analysis of (**E**) CRT and (**F**) HMGB1 expression in tumor tissues from each treatment group. Cytokine levels, including (**G**) IL-6, (**H**) IFN-γ and (**I**) TNF-α in tumor tissues from each treatment group. All data are shown as mean ± S.D. (*n* = 3), * *p* < 0.05, ** *p* < 0.01, and *** *p* < 0.001 vs. Saline.

Currently, except for its inhibitive effect on topoisomerase, EPI is also an antagonist of Forkhead box protein p3 (Foxp3), which may hold the potential to regulate the immune microenvironment in tumor tissues and thus trigger the immunogenic cell death in tumor tissues. Therefore, we first analyzed the subtype of dendric cells (DC) in tumor-draining lymph nodes through flow cytometry. As indicated in Figure 4A,C, in contrast with the saline group (2.7%), CD11+CD86+ T cells in the V+E@Gel group increased to 12%, suggesting the maturation of DC cells. Additionally, infiltration of CD8+ T lymphocytes in the V+E@Gel group (Figure S4) was also detected in immunofluorescence analysis of tumor slices. Activation markers (CD40L) and cytotoxic activity (Granzyme B, IFN-γ, and TNF-α) of these infiltrated CD8+ lymphocytes were further evaluated by immunohistochemical or immunofluorescence analysis (Figure S5). In contrast with the low positive areas in the control group, strong fluorescence signals or positive areas could be detected in the V+E@Gel group, indicating the immunogenic cell death of tumor tissues. The expression of calreticulin (CRT) and high mobility group protein (HMGB1) in tumor slices detected by immunofluorescence (Figure 4B,C,E,F) further verified our speculation as the expression of CRT and HMGB1 in V+E@Gel group were both significantly upregulated by 9-fold and 6-fold, respectively, in comparison with the saline group. The cytokine level in serum (Figure 4G–I) also proved the ICD in tumor tissues. In contrast with the saline group, the level of IL-6, IFN-γ, and TNF-α in the V+E@Gel group were enhanced by 2-, 1.6-, and 4-fold, respectively, demonstrating the strong and robust immune reaction in tumor tissues.

**Figure 5:**
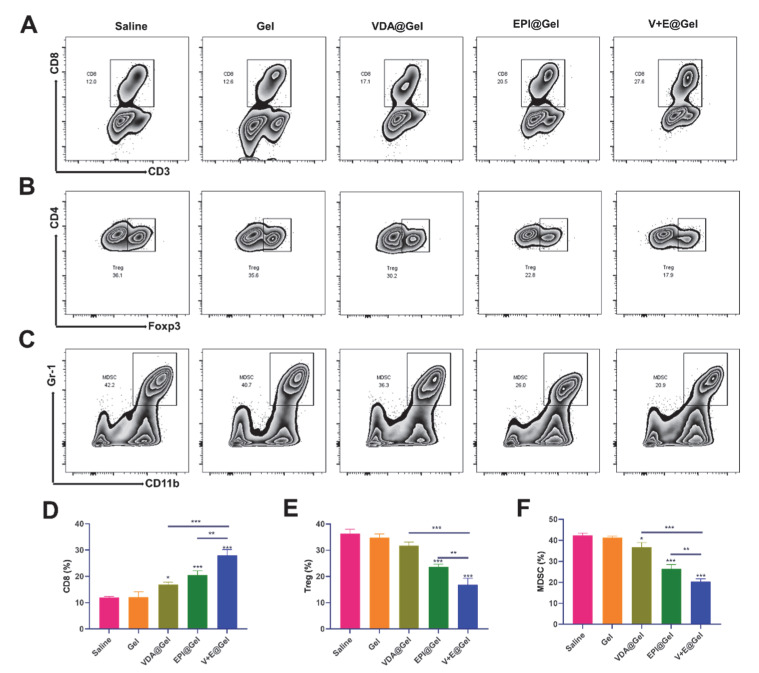
V+E@Gel induced robust immune reaction in tumor tissues. (**A**–**C**) Flow cytometric analysis and (**D**–**F**) quantitative analysis of CD8^+^ T cells after gating on CD3^+^CD8^+^ cells, Treg cells after gating on CD4^+^FOXP3^+^ cells, and MDSCs after gating on Gr-1^+^CD11b^+^ cells. All data are shown as mean ± S.D. (*n* = 3). * *p* < 0.05, ** *p* < 0.01, and *** *p* < 0.001 vs. Saline.

The specific immune response and the tumor immune microenvironment in tumor tissues were further verified by flow cytometric analysis. As shown in Figure 5A,D CD8+ T-lymphocytes in tumor tissues from the V+E@Gel group increased by 1.3-fold in comparison with the saline group. Moreover, due to the regulation effect of CA4P on the tumor immune microenvironment, CD8+ T-lymphocytes in tumor tissues from the V+E@Gel group were 61% and 35% more than that from VDA@Gel, and EPI@Gel group, respectively. This phenomenon was further proved by the flow cytometric analysis of the subtype of Treg and MDSCs cells. As indicated in Figure 5B,C,E,F, Treg and MDSCs cells in tumor tissues from the V+E@Gel group were both decreased by ~50% in comparison with the saline group, suggesting the immune surveillance in tumor tissues was effectively relieved. Further quantitative analysis of the number of total CD4+ and FOXP3+ CD4 T cells also proved the above speculation (Figure S6). Additionally, though the activation of specific immune response by VDA@Gel was not so obvious (12.6% CD8+ T-lymphocytes), its effect on regulating the Treg and MDSCs cells was strong as the Treg and MDSCs cells in tumor tissues from VDA@Gel group were decreased by 16.3% and 14%, respectively, in comparison with the saline group. Overall, due to the sustained release kinetics of V+E@Gel, its local administration may effectively relieve the immune surveillance in tumor tissues and thus induce robust and long-lasting ICD, which thus may be applicable in the clinical treatment of breast cancer.

### 3.4. Biosafety and Toxicity of V+E@Gel

The biocompatibility of the V+E@Gel group was thus preliminarily studied through histological analysis of mice’s major organs and routine biochemical examination. It could be observed in Figure S7, after s.c. injection for 5, 15 and 30 days, V+E@Gel in BALB/c did not induce obvious pathological changes in major organs, including lung, liver, spleen, kidney and heart. Moreover, s.c. injection of V+E@Gel in BALB/c did not cause any acute toxicity on mice liver, as the level of biochemical indices, including ALT, TP and AST (Figure S8), were comparable with the saline group, and all maintained normal ranges. Additionally, its toxicity on the kidney was also low, considering that the level of CREA, UA and BUN (Figure S8) were all in normal ranges on days 5, 15 and 30. Therefore, this biocompatible V+E@Gel with great potential for regulating tumor immune microenvironment and inhibiting tumor growth and metastasis may be applied in the treatment of breast cancer.

## 4. Conclusions

In this work, a classic injectable thermosensitive PLGA-PEG-PLGA copolymer was used to construct a platform to co-deliver VDA and EPI for inducing immunogenic cell death of cancer cells through targeting the tumor immune microenvironment. Its antitumor investigation on 4T1 tumor-bearing mouse models demonstrated that local administration of injectable V+E@Gel might effectively inhibit the proliferation of cancer cells and prolong the survival rate of 4T1 tumor-bearing mouse models. Histological analysis further proved that V+E@Gel could effectively inhibit tumor angiogenesis and metastasis by down-regulating the expression of CD34, CD31, MTA1 and TGF-β. Moreover, due to the sustained release kinetics of V+E@Gel, its local administration may effectively relieve the immune surveillance in tumor tissues and thus induce a robust and long-lasting specific antitumor immune response. However, it should be noted that the administration sequence of VDA and EPI is another critical element that needs to be optimized in this hydrogel formulation, as Ryszard Smolarczyk et al. mentioned recently in their work that the antitumor effect of the combination of brachytherapy and VDA on melanoma tumors may be abolished if the administration sequence was reversed [50]. Overall, this biocompatible V+E@Gel holds great potential in regulating tumor immune microenvironment and inhibiting tumor growth and metastasis, which opens up a new avenue for malignant cancer treatment.

## Data Availability

The data presented are available from the corresponding author upon request.

## Supplementary Materials

The following supporting information can be downloaded at https://www.mdpi.com/article/10.3390/pharmaceutics14091809/s1, Figure S1: Representative photos of mice treated with different formulations; Figure S2: The change in mice body weight from different treatment groups during 15 days evaluation; Figure S3: The change in mice body temperature from different treatment groups during 15 days evaluation; Figure S4: Infiltration of CD8+ T lymphocytes in tumor slices from different treatment groups; Figure S5: Histological analysis of activation marker and cytotoxic activity marker in tumor slices from different treatment groups; Figure S6: Treg cell expression analysis; Figure S7: H&E staining of mice major organs at 5 d, 15 d and 30 d post s.c. injection of V+E hydrogel; Figure S8: Level of mice biochemical indices at 5 d, 15 d and 30 d post s.c. injection of V+E hydrogel.
