# Supplementary material for: Vascular Disruptive Hydrogel Platform for Enhanced Chemotherapy and Anti-Angiogenesis through Alleviation of Immune Surveillance
Source: Pharmaceutics. 2022 Aug 28;14(9):1809. doi: 10.3390/pharmaceutics14091809 (PMC9505154; doi:10.3390/pharmaceutics14091809)
Supplement: Supplementary file 1 [file pharmaceutics-14-01809-s001.zip › pharmaceutics-1821304-supplementary.pdf]

Supporting information

Vascular Disruptive Hydrogel Platform for Enhanced Chemotherapy and Anti-Angiogenesis Through Alleviation of Immune Surveillance

Fasheng Li[1#], Xinmei Shao[2#], Dehui Liu[1], Xiaogang Jiao[1], Xinqi Yang[1], Wencai Yang[3*], Xiaoyan Liu[2*]

[1] Department of Imaging, The Fifth Affiliated Hospital of Jinan University, Jinan University, Heyuan, China

[2] Department of Neurology, The Fifth Affiliated Hospital of Jinan University, Jinan University, Heyuan, China

[3] Department of Interventional, The Fifth Affiliated Hospital of Jinan University, Jinan University, Heyuan, China

[#]These authors contributed equally to this work

correspondence: wcyang789@jnu.edu.cn (W.Y.) and liuxiaoyan2007@jnu.edu.cn (X.L.)

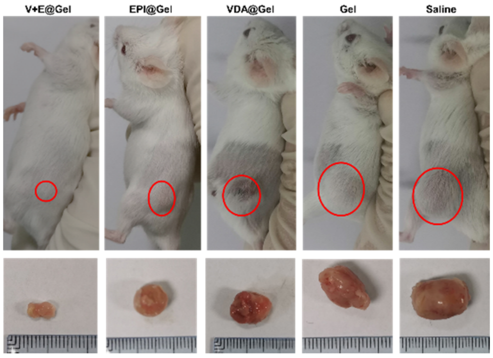

Figure S1. Representative photos of mice and tumors treated with different formulations, tumor from each group was circled by red line.

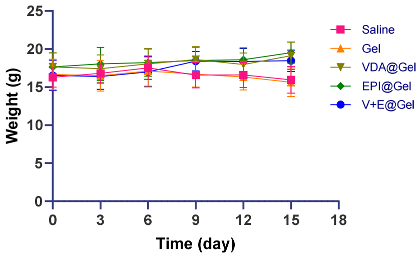

Figure S2. The change of mice body weight from different treatment groups during 15 days evaluation. All data are shown as mean ± S.D. (n = 5).

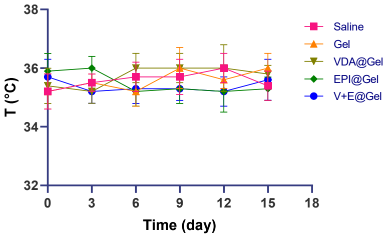

Figure S3. The change of mice body temperature from different treatment groups during 15 days evaluation. All data are shown as mean ± S.D. (n = 5).

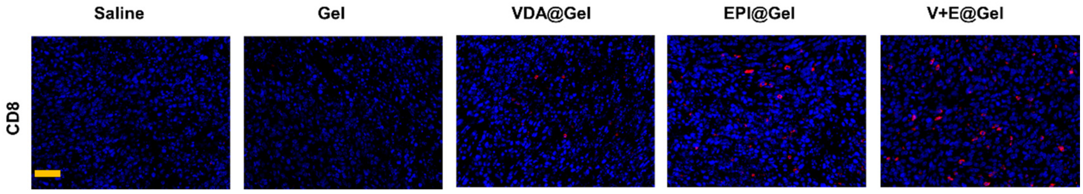

Figure S4. Infiltration of CD8+ T lymphocytes in tumor slices from different treatment groups, scale bar = 50 μm.

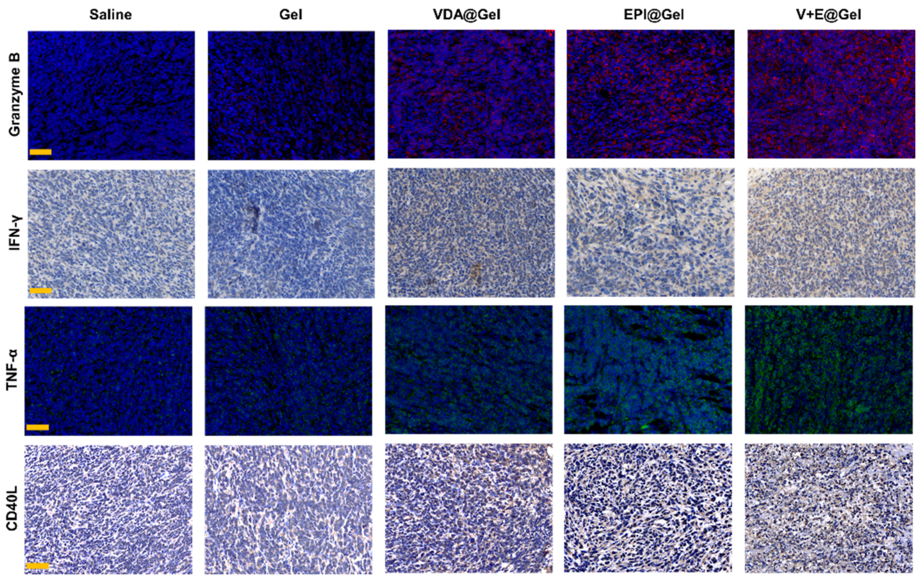

Figure S5. Histological analysis of activation marker (CD40L) and cytotoxic activity marker (Granzyme B, IFN-γ, TNF-α) in tumor slices from different treatment groups, scale bar = 50 μm.

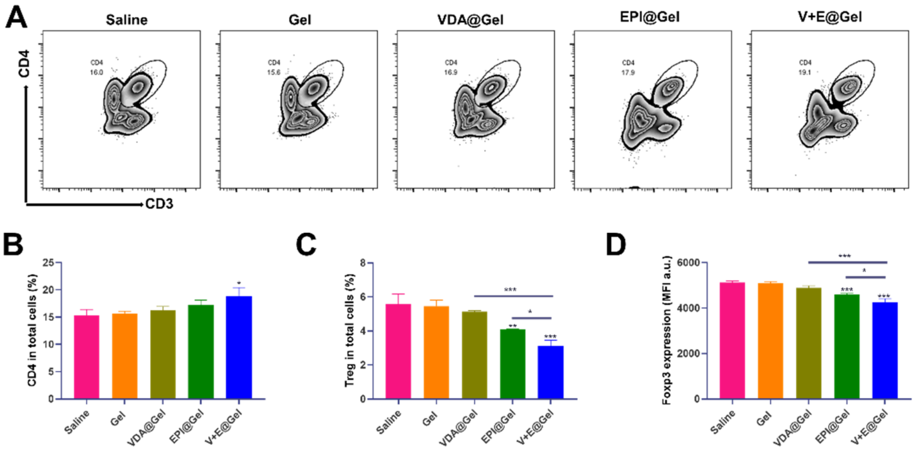

Figure S6. Treg cells expression analysis. (A)Flow cytometric analysis of $CD3^+$ $CD4^+$ T cells after gating on $CD45^+$ cells; (B) Percentage of $CD4^+$ cells and (C) Treg cells in total cells; (D) Quantitative analysis of Foxp3 expression of tumor cells by flow cytometry. All data are shown as mean ± S.D. (n = 3), **p* < 0.05, ***p* < 0.01, and ****p* < 0.001 vs. Saline group.

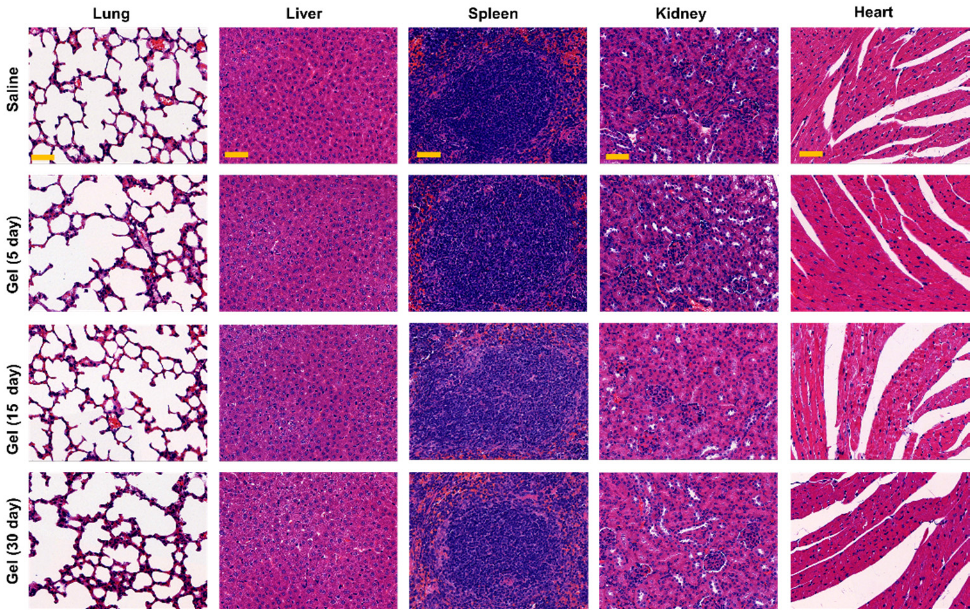

Figure S7. H&E staining of mice major organs at 5 d-, 15 d- and 30 d-post s.c. injection of V+E hydrogel, scale bar = 50 μm.

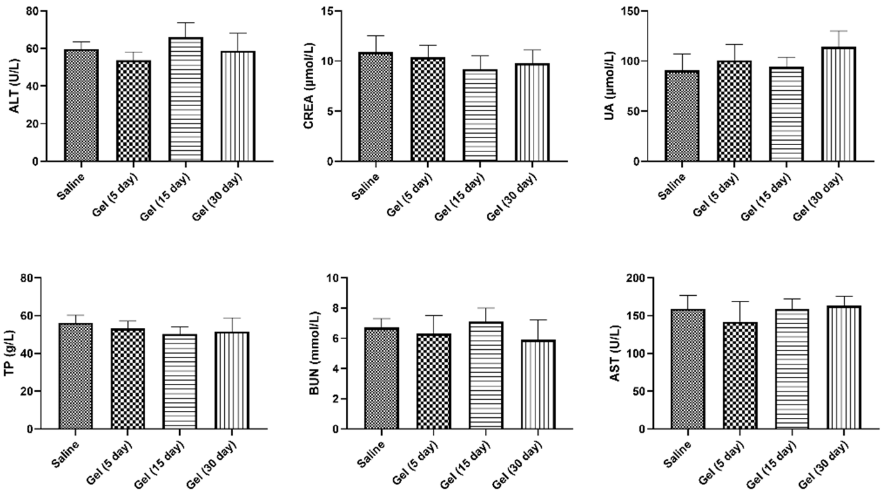

Figure S8. Level of mice biochemical indices at 5 d-, 15 d- and 30 d-post s.c. injection of V+E hydrogel. All data are shown as mean ± S.D. (n = 3).
